# Massive caffeine overdose with extremely high blood caffeine concentration and prolonged toxidrome

**DOI:** 10.1016/j.toxrep.2025.102177

**Published:** 2025-11-27

**Authors:** Yuka Okazaki, Suguru Hitomi, Shigemasa Taguchi, Ryoko Kyan, Tomohiro Yoshizawa, Yoshito Kamijo, Tomoki Hanazawa, Kazuya Kiyota

**Affiliations:** a Advanced Emergency and Critical Care Center, Saitama Red Cross Hospital, Saitama City, Saitama, Japan; b Department of Clinical Toxicology, Saitama Medical University Hospital Clinical Toxicology Center, Moro Hongo 38, Moroyama town, Iruma-gun, Saitama, Japan

**Keywords:** caffeine, overdose, prolonged, toxidrome, case report

## Abstract

The easy availability of caffeine has led to an increased risk of ingesting toxic levels in recent years. Although several studies reported severe and fatal cases of massive caffeine poisoning, few cases of caffeine overdose with a protracted toxidrome have been described. We present the case of a 37-year-old man with a massive caffeine overdose. He presented with altered consciousness and non-sustained ventricular tachycardia and was intubated and placed on continuous venovenous hemodiafiltration. The patient was extubated but had to be reintubated on day 4 because of altered consciousness, tachypnea, and tachycardia. His renal and hepatic dysfunction worsened, requiring three sessions of hemodialysis. The patient was eventually extubated on day 8, without worsening of symptoms. The present case illustrates high caffeine concentration as a cause of prolonged toxidrome with hepatic and renal damage.

## Highlights

• Few cases of caffeine overdose with a protracted toxidrome have been described.
• A 37-year-old man with a caffeine overdose caused prolonged toxidrome whose caffeine concentration was high.
• The present case illustrates high caffeine concentration as a cause of prolonged toxidrome with hepatic and renal damage.

## 1 Introduction

Caffeine is a natural alkaloid found in tea leaves, coffee and cacao beans, and kola nuts [1]. Caffeinated supplements and energy drinks can be easily obtained from brick-and-mortar and online stores and vending machines. The wide availability of caffeine is associated with an increased risk of ingesting toxic levels of caffeine, which has been observed in recent years, [2] with several fatal cases of massive caffeine overdose reported to date [2], [3].

In cases with a relatively mild caffeine overdose, the symptoms improve in a short time due to the relatively short elimination half-life of approximately 3–6 h [4]. However, few studies reported massive caffeine intoxication with a protracted toxidrome [5] and the characteristics of the prolonged clinical course and the mechanism underlying massive caffeine poisoning remain unknown. Understanding the course of prolonged toxidrome should serve as a reference for monitoring the progress of symptoms in patients with massive caffeine poisoning. Here, we report the case of a patient with prolonged toxidrome following massive caffeine intoxication. We present the clinical progression in relation to the caffeine concentrations to elucidate factors contributing to the protracted presentation of symptoms, with comparison to previously reported cases.

## 2 Case report

**Fig. 1:**
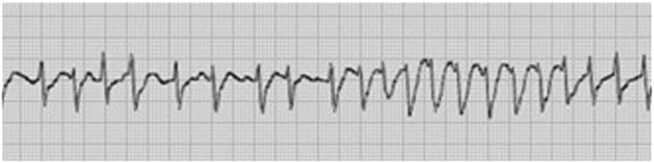
Premature ventricular contraction and non-sustained ventricular tachycardia on admission.

A 37-year-old man attempted suicide by ingesting a total of 40 g of caffeine tablets (0.53 mg/kg). He had been prescribed vortioxetine, amoxapine, eszopiclone, and levomepromazine from a psychiatric clinic for depression, but denied overdosing on any medications other than caffeine. Three hours later, the patient arrived in the emergency department. At admission, the Glasgow Coma Scale score was 10 (E3V2M5) and the patient was excited. The vital signs were as follows: respiratory rate, 16 breaths/min; peripheral arterial oxygen saturation, 80 % under oxygenation at 10 L/min; heart rate, 159 beats/min; and blood pressure, 116/95 mmHg. He was 168 cm tall and weighed 76.5 kg. He did not complain of nausea or vomit. Electrocardiography revealed premature ventricular contractions and non-sustained ventricular tachycardia (Fig. 1). Blood tests revealed the following: white blood cell count, 32740/μL; creatine phosphokinase (CK), 564 IU/L; creatinine, 1.14 mg/dL; and potassium, 3.0 mmol/L. Arterial blood gas analysis revealed respiratory and metabolic acidosis (pH 7.033; PaCO_2_, 50.3 mmHg; PaO_2_ 95.4 mmHg; HCO_3_^−^, 13.1 mmol/L; and lactate, 11.85 mmol/L). Caffeine blood concentration could not be measured during the hospitalization of the patient.

**Fig. 2:**
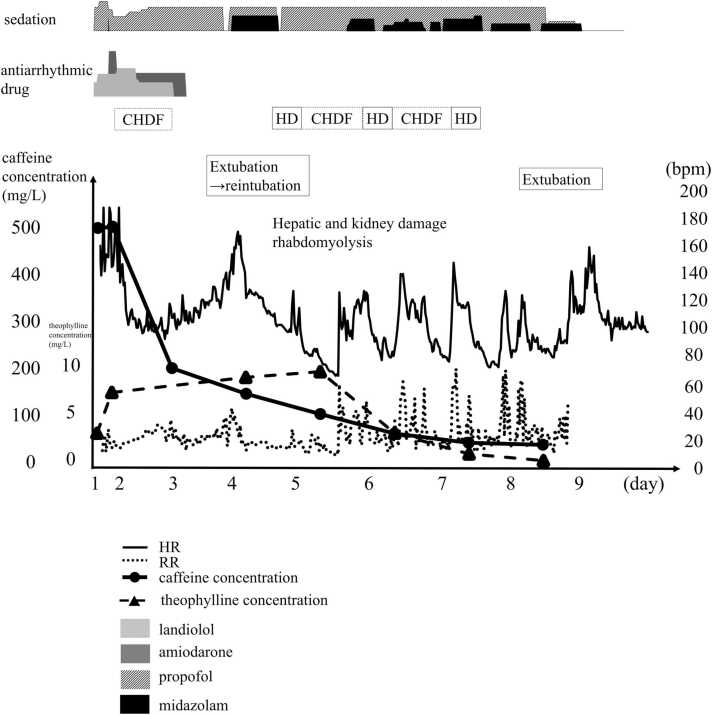
Toxidromes and treatment

The patient was intubated, and landiolol was intravenously administered for arrhythmia. However, the limited effect of landiolol required the subsequent administration of amiodarone. Additionally, hypokalemia was corrected with standard treatment. Simultaneously, 50 g activated charcoal and 50 g of 68 % magnesium citrate were administered through a gastric tube. Arrhythmia temporarily improved to normal sinus rhythm following landiolol treatment. However, due to worsening acidosis and recurrent tachycardic arrhythmia, continuous venovenous hemodiafiltration (CVVHDF) was initiated approximately 14.5 h after the overdose, based on the possibility that the patient would not be able to tolerate the high blood flow rate of hemodialysis because of hemodynamic instability. CVVHDF was performed at a blood flow rate of 100 mL/min, a dialysate flow rate of 1.0 L/h, and an ultrafiltration rate of 1.5 L/h. CVVHDF was effective in improving tachycardic arrhythmia and metabolic acidosis. The heart and respiratory rates and drug dosages are shown in Fig. 2. Both landiolol and amiodarone were discontinued on day 3, and arrhythmia did not return. Moreover, CVVHDF and sedation were terminated on day 3 due to the improvement of symptoms and the ability of the patient to communicate, suggesting an improvement in the caffeine toxidrome.

Although tachycardia and tachypnea reemerged the same day, pain associated with intubation was considered the underlying cause. The patient was extubated but developed tachypnea, tachycardia, and altered consciousness after extubation. No significant increase was noted in inflammation, nor were epileptic discharges observed on the EEG. He was not delirious, considering he did not show any diurnal variation. He did not have bradycardia, and the chances of propofol infusion syndrome were unlikely. Therefore, we suspected persistent caffeine toxidrome. The patient was sedated and reintubated 1.5 h after extubation. The renal and liver dysfunction worsened on day 5. The elevated serum CK level of 32,230 IU/L on day 3, 25,552 IU/L on day 4, and no improvement as 25,844 IU/L on day 5; these changes were accompanied by worsening renal function. The theophylline concentration, which was measured to assess caffeine metabolism, increased from 7.7 mg/L on day 2–9.3 mg/L on day 4 and 9.9 mg/L on day 5 (Fig. 2). Worsening of rhabdomyolysis and renal dysfunction was suspected. Tachypnea, tachycardia, and agitation were also observed after placing the patient in shallow sedation on day 5; prolonged caffeine toxidrome was considered as the cause. Three sessions of hemodialysis were performed, on days 5–7, each session lasting 3 h, alongside CVVHDF. Each day, sedation was interrupted for the assessment of toxidrome. However, the patient repeatedly became tachypneic and tachycardic following the discontinuation of all sedation. Sedation with propofol alone was insufficient, and the addition of midazolam resolved the tachycardia and agitation. After 3 days of hemodialysis, the patient was extubated on day 8, although tachypnea and tachycardia remained. The patient did not need sedation after extubation. No further worsening of symptoms was observed, and the patient was transferred to a psychiatric hospital on day 13.

After discharge, the serum concentrations of caffeine and theophylline, a caffeine metabolite, were measured in available blood samples using liquid chromatography-tandem mass spectrometry (SCIEX, Framingham, MA, USA) [6]. As shown in Fig. 2, the serum caffeine and theophylline concentrations were 490 mg/L and 3.4 μg/mL, respectively, at admission. The serum caffeine concentration was 120.9 mg/L on day 4 when the patient was reintubated. However, no further increases in serum caffeine concentrations were observed beyond day 4. Specifically, the serum caffeine concentrations were 75.8, 32.6, and 11.9 mg/L on days 5, 6, and 7, respectively, during hemodialysis with repeat CVVHDF.

## 3 Discussion

As illustrated in the present case, the ingestion of a massive amount of caffeine resulted in markedly elevated serum caffeine levels with prolonged caffeine toxidrome. Several studies reported cases with massive oral ingestion of caffeine [3], [5], [7], [8], [9], [10], [11]. Holstege et al. reported a patient with a serum caffeine concentration of 405 mg/L who required one HD and intubation for 17 days owing to persistent symptoms [7]. Of two cases with a caffeine blood concentration of 200–250 mg/L, one required three HD sessions and another required only one HD session [8], [9]. In all but one case, a single HD session was sufficient for cases with a caffeine concentration of < 200 mg/L [3], [5], [10], [11]. Blood caffeine concentrations above 400 mg/L may predict the prolongation toxidrome, considering the previous cases. In the present case, the serum caffeine concentration was 120.9 mg/L on day 4, when the first extubation attempt took place, indicating that the tachycardia, tachypnea, and altered consciousness were symptoms of caffeine intoxication. Overdose due to excessively high doses of caffeine should be handled with the expectation of extensively prolonged intoxication symptoms, even if intervention for arrhythmias and acidosis may not be necessary. The availability of instrumentation to determine blood caffeine concentrations should facilitate the prediction of symptomatic progression in patients with massive caffeine overdose.

The mechanism underlying the prolonged course of caffeine poisoning in patients with severe symptoms and high blood caffeine concentrations is not well known. Sato et al. proposed intestinal reabsorption as a cause of the prolonged symptoms in such cases [5]. Although gastrointestinal decontamination was performed in our patient, it is possible that caffeine might have been reabsorbed. The patient did not develop ileus during his hospital stay. However, caffeine intoxication was noted with troublesome formation in the stomach [12]; endoscopy may have been useful in evaluating pharmacobezoars. In light of the studies investigating the pharmacology of caffeine, we propose two other factors as potential causes of the protracted toxidrome observed in our patient. First, the patient ingested a very large dose of caffeine (40 g) and the initial blood caffeine concentration was extremely high (492.7 mg/L). Caffeine is primarily metabolized to 1,7-dimethylxanthine (i.e., paraxanthine) in the liver via the cytochrome P450 (CYP) isozyme CYP1A2, which causes the 3-demethylation of caffeine [13]. The elimination half-life of caffeine is variable, with an average of 3–6 h in healthy humans [4]. However, the clearance of caffeine may be substantially reduced with increasing doses of caffeine [14], due to the saturation of CYP1A2 [15]. The patient did not receive any other medication that could interfere with CYP 1A2. In the present case, after hemodialysis for 3 days, the serum caffeine concentration declined to below 50 % of the admission concentration. The clearance of caffeine was reduced as well. The second potential cause of the protracted toxidrome is the direct inhibition of caffeine metabolism and urinary excretion by the caffeine poisoning. In the present case, hepatic and renal failure worsened on day 5. Rhabdomyolysis, which is a potential outcome of caffeine overdose [16] that can lead to acute renal injury, was worsened at the peak on day 5. The serum caffeine concentration declined from 120.9 mg/L on day 4–75.8 mg/L on day 5 without hemodialysis. The elimination of caffeine primarily occurs via excretion in urine (85 %–88 %) [17]. Hepatic and renal dysfunction may lead to the prolonged clinical course observed in cases of massive caffeine poisoning.

In the present case, we also measured theophylline levels, as a reference for caffeine metabolism. The maximum theophylline concentration was 9.9 mg/L, and we do not predict that the symptoms were aggravated by the metabolic products. However, we did not measure the levels of paraxanthine, another caffeine metabolite. Caffeine is absorbed after ingestion, and approximately 84 % of the ingested caffeine is metabolized to paraxanthine, whose antagonistic effect on adenosine receptors is approximately twice as strong as that of caffeine. Yamazaki et al. reported that the increased paraxanthine and CK levels 6 h after hemodialysis in patients with high blood caffeine concentrations might prolong the symptoms of caffeine intoxication despite the decrease in caffeine concentrations after hemodialysis [18]. It remains possible that paraxanthine caused the prolongation of caffeine toxidrome in the present case.

Our patient might have had the highest caffeine concentration among the reported survivors. Caffeine concentrations above 80 mg/L are lethal [19]. In a study by Jones et al., which included 51 cases with caffeine overdose as the main cause of death, the mean postmortem blood caffeine concentration was 187 ± 96 mg/L [20]. Yoshizawa et al. reported that the median blood caffeine concentration was 240.6 ± 129.6 mg/L in patients with severe caffeine poisoning [21]. Our patient survived, although the blood caffeine concentration far exceeded the reported lethal levels. Arrythmia often determines the prognosis of patients with caffeine poisoning; however, factors associated with arrhythmogenicity and therapeutic response remain unknown. Arrythmia might be refractory in our patient, given its recurrence after the initial improvement following the administration of landiolol and amiodaron. Further studies are warranted to more clearly characterize the individual differences in mortality and the efficacy of antiarrhythmic drugs in patients with caffeine overdose.

Regarding the choice of blood purification, CVVDHF, and not hemodialysis, was initially used because of circulatory instability. The improvement of symptoms, such as arrythmia, in addition to the decline in serum caffeine concentration from 492 to 178 mg/L indicate the efficacy of CVVDHF in the present case. However, the serum caffeine concentration of 178 mg/L was still high and could lead to the need for reintubation and the development of organ damage. More effective blood purification approaches could have been performed in high-flow CVVDHF. Moreover, the determination of caffeine concentration at admission would have guided the decision to continue CVVDHF and avoid unnecessary early extubation.

Herein, we observed the following: The patient was not experiencing tachypnea when he arrived at the hospital, potentially because of an overdose of other medications in addition to caffeine, but the patient did not disclose this information. Withdrawal symptoms from the patient’s original medication may have contributed to the tachycardia and other symptoms.

## 4 Conclusion

As illustrated in the present case, an extremely high caffeine concentration in serum might predict a longer-than expected caffeine toxidrome.

## CRediT authorship contribution statement

**Kazuya Kiyota:** Writing – original draft. **Yoshito Kamijo:** Writing – review & editing. **Tomohiro Yoshizawa:** Writing – review & editing. **Tomoki Hanazawa:** Writing - review & editing. **Ryoko Kyan:** Writing – review & editing. **Shigemasa Taguchi:** Writing – original draft. **Suguru Hitomi:** Writing – original draft. **Yuka Okazaki:** Writing – original draft, Visualization.

## Patient consent statement

Patient consent has been obtained.

## Funding sources

This research did not receive any specific grant from funding agencies in the public, commercial, or not-for-profit sectors.

## Declaration of Competing interest

The authors declare that they have no known competing financial interests or personal relationships that could have appeared to influence the work reported in this paper.

## Data Availability

No data was used for the research described in the article.

## References

[1] Cappelletti S., Daria P., Sani G., Aromatario M. (2015). Caffeine: Cognitive and physical performance enhancer or psychoactive drug?. Curr. Neuropharmacol..

[2] Ishigaki S., Fukasawa H., Kinoshita-Katahashi N., Yasuda H., Kumagai H., Furuya R. (2014). Caffeine intoxication successfully treated by hemoperfusion and hemodialysis. Intern. Med..

[3] Yasuda S., Hisamura M., Hirano T., Kukihara Y., Kodama K., Konishi K. (2021). Caffeine poisoning successfully treated by venoarterial extracorporeal membrane oxygenation and emergency hemodialysis. Acute Med. Surg..

[4] Arnaud M.J. (2011). Pharmacokinetics and metabolism of natural methylxanthines in animal and man. Handb. Exp. Pharmacol..

[5] Satoshi S., Akihito T., Katsuhiro O., Keiko S., Hitoshi N., Takahiro I. (2021). Reelevated blood caffeine level due to intestinal reabsorption in a patient with caffeine intoxication. J. Jpn. Soc. Intensive Care Med..

[6] Hanazawa T., Kamijo Y., Yoshizawa T., Usui K. (2021). Rapid measurement of serum caffeine concentrations in acuteclinical settings. Toxicol. Commun..

[7] Holstege C.P., Hunter Y., Baer A.B., Savory J., Bruns D.E., Boyd J.C. (2003). Massive caffeine overdose requiring vasopressin infusion and hemodialysis. Journal Toxicology Clinical Toxicology.

[8] Nishimura Y., Iwamura T., Koami H., Yamashita T., Nakashima A., Inoue S. (2013). A case of caffeine overdose successfully treated with hemodialysis. Nihon Kyukyu Igakukai Zasshi.

[9] Kohl B.A., Kaur K., Dincher N., Schumann J., Carachilo T., Komurek C. (2020). Acute intentional caffeine overdose treated preemptively with hemodialysis. Am. J. Emerg. Med..

[10] Kodate A., Yoshimura S., Watanabe A., Fujimi S., Yoshimura D., Hayashi A. (2019). A case of acute caffeine poisoning in a patient showing a re-elevated caffeine level. Jpn. J. Clin. Toxicol..

[11] Tajima E., Tominaga H., Takahashi H., Yoshimura K., Hattori J. (2019). Successful treatment of acute caffeine intoxication with hemodialysis. J. Jpn. Soc. Dial. Ther..

[12] Takahashi A., Maruhashi T., Maruki H., Kim M., Oi M., Yoshimura K. (2025). Caffeine poisoning with a re-elevated blood caffeine concentration after termination of continuous hemodiafiltration despite endoscopic gastric lavage for a drug mass formed in the stomach：A case report. Jpn J. Clin. Toxicol..

[13] Willson C. (2018). The clinical toxicology of caffeine: a review and case study. Toxicol. Rep..

[14] Hirose M., Hirakawa A., Niwa W., Higashiguchi T., Tajima K., Kato T. (2021). Acute drug poisoning among adolescents using over-the-counter drugs: current status. Yakugaku Zasshi.

[15] Mandel H.G. (2002). Update on caffeine consumption, disposition and action. Food Chem. Toxicol..

[16] Chiang W.F., Liao M.T., Cheng C.J., Lin S.H. (2014). Rhabdomyolysis induced by excessive coffee drinking. Hum. Exp. Toxicol..

[17] Bonati M., Latini R., Galletti F., Young J.F., Tognoni G., Garattini S. (1982). Caffeine disposition after oral doses. Clin. Pharmacol. Ther..

[18] Yamazaki Y., Kaizaki-Mitsumoto A., Sato M., Inoue Y., Miyamoto K., Suzuki K. (2024). Simultaneous analysis of caffeine and paraxanthine provides potentially useful indexes in the treatment of acute caffeine intoxication. J. Toxicol. Sci..

[19] Kaplan G.B., Greenblatt D.J., Ehrenberg B.L., Goddard J.E., Cotreau M.M., Harmatz J.S. (1997). Dose-dependent pharmacokinetics and psychomotor effects of caffeine in humans. J. Clin. Pharmacol..

[20] Jones A.W. (2017). Review of caffeine-related fatalities along with postmortem blood concentrations in 51 poisoning deaths. J. Anal. Toxicol..

[21] Yoshizawa T., Kamijo Y., Hanazawa T., Usui K. (2021). Criterion for initiating hemodialysis based on serum caffeine concentration in treating severe caffeine poisoning. Am. J. Emerg. Med..

